# Leveraging remote learning during the Covid‐19 pandemic to enhance student understanding of biodiversity

**DOI:** 10.1002/ece3.8729

**Published:** 2022-03-21

**Authors:** Christine L. Weilhoefer, Sarah Schmits

**Affiliations:** ^1^ Departments of Biology and Environmental Studies University of Portland Portland Oregon USA; ^2^ Department of Biology University of Portland Portland Oregon USA

**Keywords:** ecology education, environmental perceptions, latitudinal diversity gradient, nature‐based educational experience, online education, undergraduate education

## Abstract

We evaluated whether individual nature‐based ecological (NBE) study used in tandem with group collaboration enhanced undergraduate student understanding of ecological concepts and pro‐environmental perceptions. In response to the Covid‐19 pandemic, we developed a multiweek unit on the latitude diversity gradient (LDG) for fully online instruction that leveraged the unique situation of students learning in disparate geographic locations. Student understanding of the LDG and pro‐environmental perceptions were assessed with surveys administered both pre‐ and post‐activity in an introductory‐level biology laboratory course. Student understanding of the geographic location where biodiversity is the highest was high prior to the start of the laboratory unit and exhibited only a small improvement after the unit. In contrast, students’ higher order thinking around the LDG was enhanced by the lab activity. Student environmental perceptions shifted toward ecocentric views and away from anthropocentric views after the laboratory unit. The greatest gains in ecological understanding and shifts toward ecocentric viewpoints occurred in the group of students who visited their field sites most often. Our results provide further evidence as to the value of NBE for the introductory biology laboratory, even in an online learning setting. The lab unit described in this study provides a potential approach to teaching ecology in an online format that could easily be adapted to fit the needs of a particular curriculum.

## Graphical abstract

Student understanding of ecological concepts and pro‐environmental perceptions were enhanced after a multiweek field study developed for an online ecology course. Improvements were highest in the group of students who visited their field sites most often.

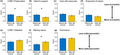

## 1 INTRODUCTION

Habitat modification, fragmentation, resource exploitation, pollution, introduction of invasive species, and climate change are transforming natural landscapes across the globe (Butchart et al., 2010; Carpenter et al., 2009; Diaz et al., 2006). Biodiversity loss is a ubiquitous consequence of this transformation, and this loss of species has been linked to changes in ecosystem function (Hooper et al., 2005; Naeem et al., 2009). While the scientific community is generally in agreement as to the relationship between human activities and biodiversity loss, biodiversity loss is often perceived by the public as a less important environmental problem compared to issues such as climate change or air pollution (Kaltenborn et al., 2016). Biodiversity is a relatively new scientific term, first used in 1985 by Walter Rosen (Medland, 2004). While most people conceptualize biodiversity as the number of species in an area, biodiversity spans organizational levels of biology from genetic diversity within a species to diversity of ecosystems (Hooper et al., 2005; Purvis & Hector, 2000; Wilson, 1988).

Studies have demonstrated that the general public has neither a strong understanding of the concept of biodiversity nor accurate ideas of the number of species within their local communities (Hunter & Brehm, 2003; Lindemann‐Mathies & Bose, 2008; Turner‐Erfort, 1997). It has been argued that the lack of understanding of the complexity inherent in the concept of biodiversity contributes to the public's perception that biodiversity loss is not an important environmental issue (Fischer & Young, 2007; Jones‐Walters & Cil, 2011; Kaltenborn et al., 2016). In addition, the public does not recognize the role of biodiversity loss in exacerbating other problems more familiar and important to them, such as habitat degradation and climate change (Novacek, 2008). More recently, there has been a focus of public awareness around biodiversity issues (e.g., Antonelli et al., 2020; Diaz et al., 2006; IPBES 2019; MEA 2005). However, despite the increased attention biodiversity has received in the media, many argue that the public's understanding of biodiversity has declined over the past few decades and is not steeped in scientific understanding (Buijs et al., 2008; Yil‐Panula et al., 2018).

Environmental education has long been cited as a tool to promote pro‐environmental perceptions and conservation as there is evidence that environmental attitudes and beliefs can be changed through education (Boeve‐de Pauw et al., 2011; Wals, 2011). However, students often fail to make the connection between environmental problems and the science of ecology (Finn et al., 2002; Roberts, 1997). The concept of ecological literacy goes beyond environmental education, integrating the understanding of nature's interrelationships with how to take actions to improve environmental health. Many researchers feel that this merging of traditional science education with environmental education is necessary to create a truly ecologically literate population (Pitt et al., 2019; Roth, 1992; Wals et al., 2014). In fact, the US EPA’s current definition of environmental education, “a process that allows individuals to explore environmental issues, engage in problem solving, and take action to improve the environment,” is evidence of this shift in attitude toward embracing scientific principles in environmental education.

Nature‐based educational (NBE) experiences, where aspects of instruction take place outside, are salient components of many environmental education programs. NBE has the potential to enhance the link between environmental education and pro‐conservation beliefs and practices by fostering a connection between the environment and personal experience (Ballantyne et al., 2001; Louv, 2006). Several studies have demonstrated that NBE enhances both environmental literacy and attitudes toward the environment (e.g., Ballantyne & Packer, 2002; McRae, 1990). NBE can be viewed as a subset of place‐based education (PBE), where the local community and environment are the starting point to teach subjects across the curriculum (Sobel, 2004). Previous studies have demonstrated the value of PBE in contributing to the understanding of global social‐ecological dynamics and in fostering ecological conservation efforts (Ardoin, 2006; Knapp, 2005; Marten‐López et al., 2020). While NBE is rooted in the ideals of PBE, the parallels between NBE and PBE may unravel at the university level where students often do not have a close connection to their local environment. This may be particularly true for first‐year students who have not yet had time to develop a connection with the environment of their college campus (Freeman et al., 2007; Jorgenson et al., 2018; Pittman & Richmond, 2008).

NBE are commonly used in ecology courses as an evidence‐based pedagogical practice shown to enhance students’ understanding of ecological concepts and practices (Carter, 1993; Hart & Nolan, 1999; Simmons et al., 2008). Field study is often a core component of ecology coursework (Boyle et al., 2007; Tewksbury et al., 2014). In *Teaching biology outside the classroom*: *Is it heading for extinction?*, Barker et al. (2005) goes so far as to state that “fieldwork is the authentic context for teaching ecology.” Beyond simple observational studies, some researchers believe ecological literacy may be further improved by an inquiry‐based approach, where students engage in a systematic process of evaluating scientific questions to support or reject hypotheses (Roberts, 1997; Zohar, 1998). Others caution that simple snapshot observational studies may not go far enough and advocate for manipulative experiments or long‐term field studies (Finn et al., 2002; Gibson et al., 1999).

Ecology classes with field components are thought to be the STEM discipline most impacted by the shift to online education in response to the Covid‐19 global pandemic (Bacon & Peacock, 2020). Many faculty reported a shift from field activities to more instructor‐centered activities (Barton, 2020). The challenge of teaching laboratory courses online was met in diverse ways; from recording laboratory demonstrations, to virtual laboratory simulations, to simple do‐at‐home experiments, and to virtual field trips (e.g., Bacon & Peacock, 2020; Youssef et al., 2020). Despite these alternatives, many instructors with field study components in their courses reported removing or planning to remove field learning outcomes (29%) or reducing or planning to reduce field learning outcomes (47%) in response to online instruction (Barton, 2020). Also, while the shift to remote learning clearly had negative impacts on pedagogy, others sought teaching and learning opportunities provided by students being located away from campus. For example, several educators advocated for using field data collected by students in diverse locations to compare across geographic regions or ecological conditions (Bacon & Peacock, 2020; Cooke et al., 2021).

In anticipation of remote learning during academic year 2020–2021, we sought opportunities to teach foundational ecological concepts that leveraged this unique learning environment. Examination of the patterns and mechanisms behind the latitude diversity gradient (LDG) seemed ideal to capitalize on the unique situation of students learning in disparate geographic locations. The LDG states that biodiversity is the highest in the tropics and decreases from equatorial to polar regions (Hillebrand, 2004; Willing et al., 2003). The LDG was one of the first gradients described in ecology and is a prevalent pattern observed across wide‐ranging types of organisms, including plants, insects, amphibians, birds, and mammals. While several mechanistic hypotheses related to ecological limits, diversification rates, and time have been proposed to explain the LDG, it is still a widely debated ecological phenomenon (Pontarp et al., 2019). The LDG integrates several other important ecological concepts, such as biodiversity, dispersal, ecological niches, and geographic range. It is therefore an ideal theme to examine multidimensional ecological comprehension.

This study aimed to evaluate whether individual NBE and PBE ecological study used in tandem with group collaboration across geographic locations enhanced student understanding of key ecological concepts, pro‐environmental perceptions, and ecological literacy. We predicted that: (1) student understanding of the LDG would increase after completion of the lab unit, (2) pro‐environment perceptions would increase upon completion of the lab unit, and (3) improvements in ecological understanding and pro‐environmental perceptions would be highest for students who visited their local field site most often.

## 2 METHODS

### 2.1 Participants

Forty‐six students across four lab sections in an introductory biology laboratory course (Bio278 Introduction to evolution and ecology laboratory) at the University of Portland in fall semester 2020 were the study population. The University of Portland is a private Catholic university in Portland, Oregon, USA with approximately 3700 undergraduate students. This course is the second course in the introductory biology series at the University of Portland and aimed at first and second‐year undergraduate students in the Biology and Environmental studies majors. Students from a variety of other majors also take this course as a prerequisite to several professional school programs or to fulfill university requirements. During fall 2020, 80% of the students were second‐year students and 60% were biology majors and 8% were environmental studies majors. Due to required at home learning during the Covid‐19 pandemic, this course was held completely online in a synchronous format. Students participated in the course from their homes in seven states (Arizona, California, Hawaii, New Jersey, Oregon, Utah, Washington), with the majority of students participating in the course from their homes in Oregon (50%) and California (22%).

### 2.2 Laboratory activity

**FIGURE 1:**
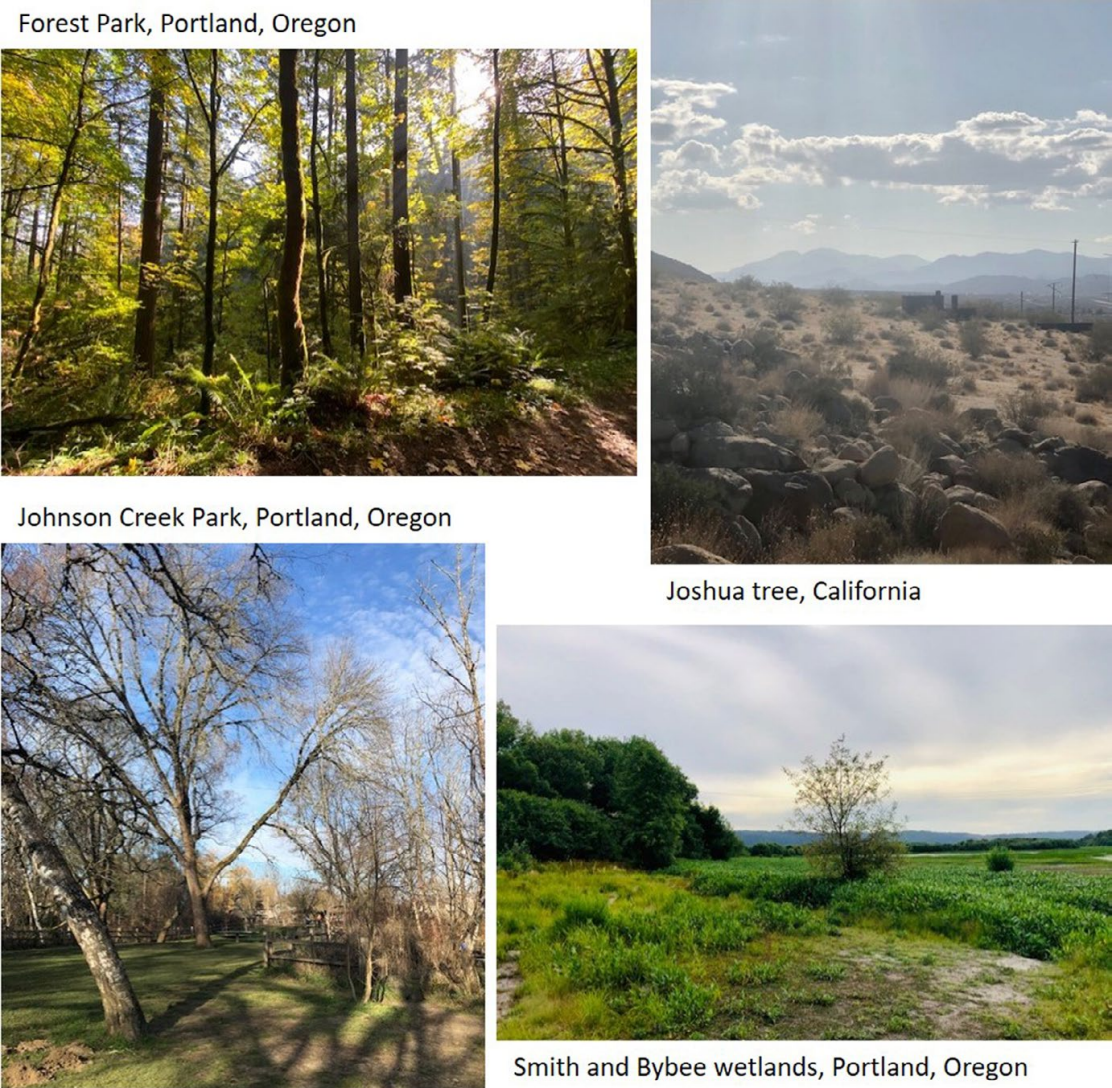
Examples of student field sites in a forested location (Forest park, Portland, Oregon), an urban wetland (Smith and Bybee wetlands, Portland, Oregon), a city park (Johnson Creek Park, Portland, Oregon), and a desert area near Joshua Tree, California

We developed a multiweek laboratory unit for fully online instruction that was both nature‐based and place‐based (see Appendix 1 for weekly schedule). For this unit, each student selected a local field site of any kind with the criteria that it was at least 30 m in length, not directly on a path or road, not in a planted lawn, and safely accessible (Figure 1). Each student was sent a field kit with supplies to conduct soil tests (pH, nitrogen, phosphorus, potassium), perform vegetation identification along transects, and identify birds. Students spent eight weeks researching the natural history of their site and conducting guided field activities to characterize soil properties, vegetation, and birds using standard ecological methods and free online species identification resources (e.g., GoogleLens, LeafSnap, PlantSnap, MerlinBird ID, SongSleuth). To increase the likelihood of accurate identification using these apps, students were asked to upload multiple photos of each plant and consult the instructor when there were any discrepancies with identification. Students calculated two biodiversity metrics for their site: species richness and Shannon diversity, for vegetation and birds separately. Students also used shared guidelines to classify the biome of their site and where their site fell along a gradient of urbanization. In the final lab activity of the biodiversity unit, students explored the veracity of the LDG. For this activity, students were put into intentional groups of four, with students being from at least three different geographic locations. Groups were tasked with graphically and statistically examining whether the class dataset conformed to the LDG by comparing the relationships between diversity and latitude found in their data with that proposed by the LDG. They used their ancillary data about site conditions and the scientific literature to posit three ecologically sound hypotheses to explain why their observations supported or refuted the LDG.

The flow of a typical three‐hour laboratory session was as follows: students were provided with a prelab activity to complete before the start of the session. Prelab activities usually involved a video demonstration of field work techniques (e.g., how to implement vegetation transects, how to characterize soil pH, key features to use when identifying birds, etc.) and a short reading related to the topic. Then, students would meet virtually with the instructor for ~30 min to one hour to go over the methodology for the lab. Students would then go to their field sites to conduct the data collection assigned for that week (Appendix 1). Finally, students would upload scans of their field notebooks, data, and submit a short reflection answering a few questions about the activity (see Appendix 2 for structure of typical weekly session). Students unable to visit their field site for any reason (e.g., stay in place orders, health issues, weather) were provided with online resources that mimicked the week's lab activity. Students with occasional schedule conflicts were given the option of watching course recordings and completing the laboratory entirely on their own, although on average fewer than two students opted for asynchronous instruction each week.

### 2.3 Ecological concept and environmental perceptions instruments

To assess whether NBE and PBE ecological study impacted student understanding of key ecological concepts and student pro‐environmental perceptions, students were given a set of identical surveys at the start and after completion of the laboratory unit. Student understanding of the LDG was examined based on their answers to a two‐question quiz administered both pre‐ and post‐ lab activity. Students were asked a single‐answer multiple choice question about where biodiversity is highest and received full credit for selecting the correct answer and no credit for selecting an incorrect answer (Appendix 3). This question aimed at testing lower‐order understanding of the LDG. Students were also asked a free response question where they described mechanisms that result in high biodiversity, testing higher‐order understanding of the LDG. For this question, each individual response was scored based on an *a priori* list of possible correct answers. An answer received a score of “1” if it demonstrated a complete understanding of the concept, “0.5” if demonstrated an incomplete understanding of the concept, and a “0” if the answer was incorrect. The scores of each of the three individual responses were summed for each student to receive a composite score between 0–3 for this question.

Students’ environmental perceptions, both before and after the laboratory unit, were measured with the 2 major environmental values (2‐MEV) scale (Bogner & Wilhelm, 1996; Bogner & Wiseman, 1999), a tool vetted and widely used in the environmental education field. The 2‐MEV scale was developed for European adolescents (ages 15–17) but has been validated across several geographic areas and cultures (Johnson & Manoli, 2011; Milfont & Duckitt, 2004) and has been successfully used in adults (Johnson & Manoli, 2011). Because participants were college‐aged (18–21 years), we used the 16‐item modified 2‐MEV scale recommended for older participants (Bogner et al., 2015; Johnson & Manoli, 2011). The 2‐MEV scale uses a series of questions to characterize a respondent's ecological values in terms of two orthogonal dimensions: an ecocentric dimension, reflecting environmental conservation and protection (preservation) and an anthropocentric dimension, reflecting the utilization and exploitation of natural resources (utilization) (Bogner et al., 2015). These higher order factors, preservation of nature (PRE) and utilization of nature (UTL), are further broken down into subscales (Table 1). The PRE factor can be further broken down into three subscales: “Intent of support”, “Care with resources”, and “Enjoyment of nature”, and the UTL factor can be broken down into two subscales: “Human dominance” and “Altering Nature”. The 2‐MEV scale items were measured using a five‐point Likert scale. The scale items were coded from 1 = “strongly agree”, 2 = “agree”, 3 = “neither agree nor disagree”, 4 = “disagree”, to 5 = “strongly disagree”. With this scoring, a score close to 1 on the PRE factor represents a strong ecocentric point of view, while a score of 5 on the PRE scale represents a less ecocentric point of view. For the UTL factor, a score close to 1 indicates an anthropocentric point of view and a score close to 5 indicates a less anthropocentric point of view. For the 2‐MEV scale, mean scores for items related to “intent of support”, “care with resources”, “enjoyment of nature”, “altering nature”, and “dominance” were calculated for each participant, as well as a mean for the PRE and UTL factors for each participant. Because several ordinal Likert items were averaged to calculate each scale item, creating interval data, univariate parametric statistical analyses were appropriate (Boone & Boone, 2012).

**TABLE 1:** Items of the 2‐MEV scale (PRE = preservation, UTL = utilization) administered pre‐ and post‐laboratory unit

Factor	Subscale	Item
PRE	Care with resources	To save energy in the winter, I make sure the heat in my room is not on too high
PRE	Care with resources	I always turn off the light when I do not need it anymore
PRE	Care with resources	I try to save water by taking shorter showers or by turning off the water when I brush my teeth
PRE	Enjoyment of nature	I would like to sit by a pond and watch dragonflies
PRE	Enjoyment of nature	I like to go on trips to places like forests away from cities
PRE	Enjoyment of nature	I like the quiet of nature
PRE	Intent of support	If I ever have extra money, I will give some to help protect nature
PRE	Intent of support	I would help raise money to protect nature
PRE	Intent of support	I try to tell others that nature is important
UTL	Altering nature	People have the right to change the environment (nature)
UTL	Altering nature	I like a grass lawn more than a place where flowers grow on their own
UTL	Altering nature	To feed people, nature must be cleared to grow food
UTL	Altering nature	Weeds should be destroyed because they inhibit the full development of useful and ornamental plants
UTL	Dominance	Building new roads is so important that trees should be cut down
UTL	Dominance	Because mosquitoes live in swamps, we should drain the swamps, and use them for farming
UTL	Dominance	People are supposed to rule over the rest of nature

### 2.4 Ancillary information

Surveys were anonymous but students reported their geographic location, how often they visited their field sites (always, most of the time, sometimes, never), and their lab section on their surveys (Appendix 3). Survey results were not examined until after semester grades were submitted.

### 2.5 Informed consent and protection of human subjects in research

The protocol and procedures for this laboratory unit, including the 2‐MEV survey, were reviewed and approved by the University of Portland's Institutional Review Board (IRB00006544) and followed according to the Helsinki Declaration of 1997 (revised in 2008). Students were given the IRB statement to read prior to the start of the laboratory unit. Subject identifying information was not recorded nor included in the manuscript.

### 2.6 Data analysis

To examine how the laboratory unit influenced ecological understanding and environmental perceptions, pre‐ and post‐survey scores were compared. There were no differences in scores for either the ecological understanding or environmental perceptions instruments between the four lab sections, therefore, data were pooled across lab sections. Scores for the entire population of participants were combined, and pre‐ and post‐comparisons were performed on these pooled data; individual student pre‐ and post‐scores were not assessed. A series of *t*‐tests were used to compare pre‐ and post‐scores for: (1) each of two questions on the ecological concept survey, (2) 2‐MEV PRE factor score, (3) 2‐MEV UTL factor score, (4) 2‐MEV intent of support item, (5) 2‐MEV care with resources item, (6) 2‐MEV enjoyment of nature item, (7) 2‐MEV altering nature item, and (8) 2‐MEV dominance item. One‐way analysis of variance (ANOVA) was used to examine the effect the number of times students visited their field site (e.g., always, most of the time, sometimes, never) had on ecological understanding and pro‐environmental perceptions after the lab activity. Prior to analysis, data were examined to ensure that the assumptions of each statistical test were met. If not, data were log‐transformed. All statistical analyses were performed in R‐version 3.6.3 (Holding the Windsock, R Development Core Team, 2018).

## 3 RESULTS

Our study had four main findings: (1) students’ understanding of the LDG was high prior to the start of the laboratory unit, (2) students’ ability to explain the mechanisms behind the LDG increased after the lab activity, with explanations shifting from single word answers to more in‐depth responses, (3) environmental perceptions shifted toward ecocentric and away from anthropocentric after the laboratory unit, and (4) the number of times a student visited their field site impacted both ability to explain the LDG and pro‐environmental perceptions.

### 3.1 Ecological concept survey

**FIGURE 2:**
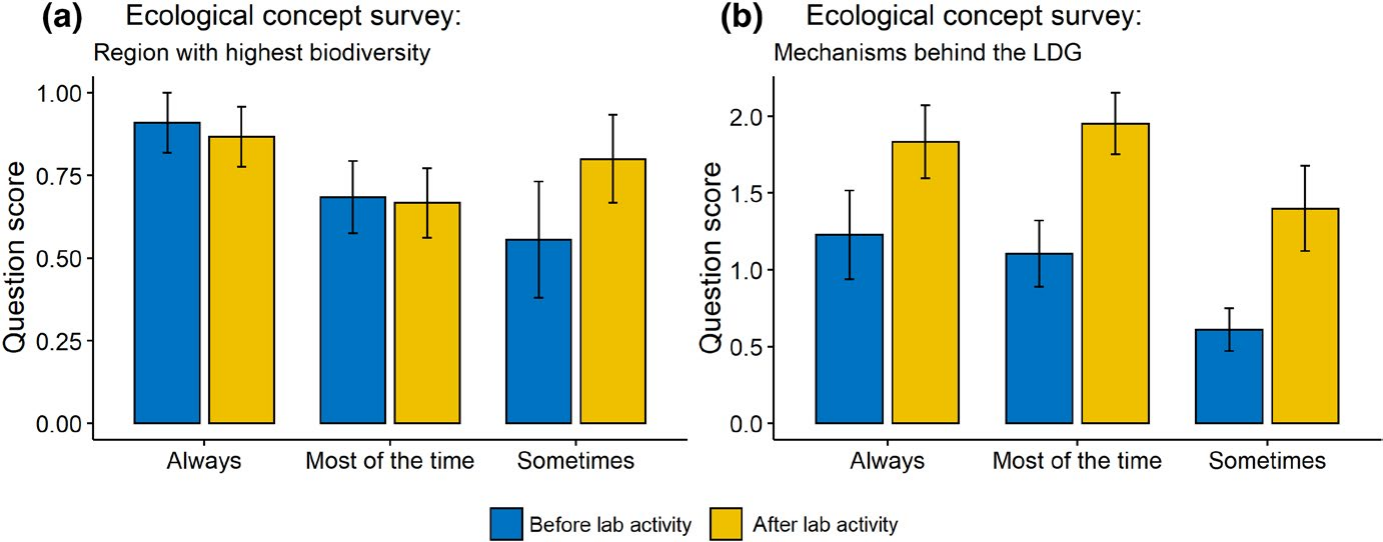
Scores of the ecological concept survey questions administered before and after the laboratory unit (mean ± standard error). For the region with the highest biodiversity, students scored “1” if they answered the question correctly and “0” if they answered the question incorrectly. For the mechanisms behind the LDG question, students scored between 0–3, depending on how many correct mechanisms they provided. A score of “3” indicates three correct answers and a score of “0” indicates no correct answers. Scores were separated based on self‐reporting of how often students visited their field site (always, most of the time, sometimes)

Students’ understanding of the LDG was assessed by a question that asked students to select the geographic location with highest species diversity and by asking students to describe three mechanisms behind this pattern. Initial understanding of the LDG concept was high, with 72% answering the quantitative question correctly on the presurvey. Postlab unit, students showed a slight but nonsignificant increase in their understanding of the concept that biodiversity is the highest in the tropics (76%). While students had a solid understanding of where biodiversity is highest before the onset of the lab unit, their ability to explain the mechanisms behind the LDG was much more limited before the lab unit (Figure 2b). Students scored 1.0 ± 0.9 (on a scale of 0 to 3, three being the highest) preactivity. Scores for postlab unit were significantly higher (mean: 1.8 ± 0.9, *t*‐test *p*‐value .00015). Answers for the presurvey question about mechanisms responsible for the LDG were often just a single word with no explanation (e.g., “weather”, “people”, “water”), while answers for the postsurvey were more in‐depth (e.g., “higher water availability”, “introduction of invasive species”, “urbanization of landscapes”).

### 3.2 Environmental perceptions survey—2 MEV

**FIGURE 3:**
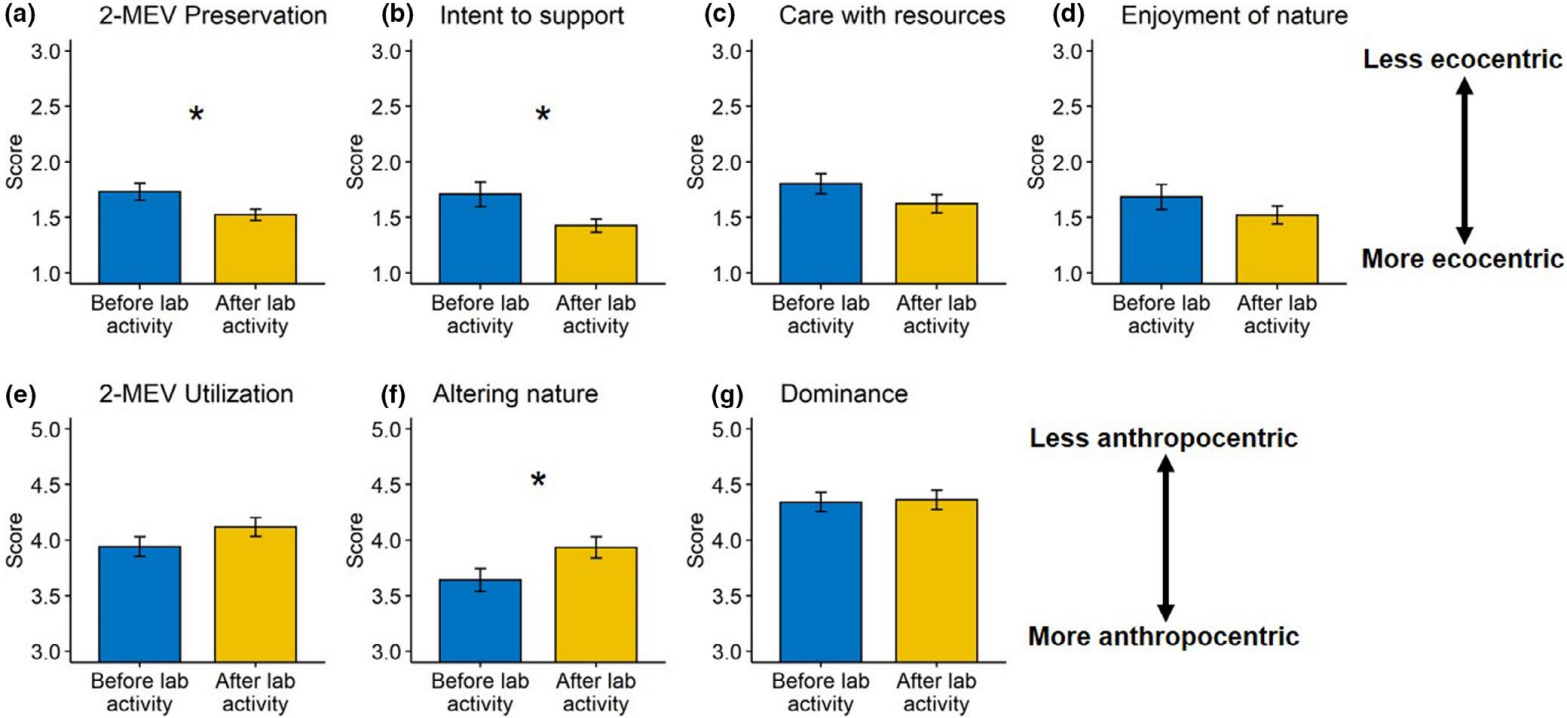
Scores on the 2‐MEV environmental perception instrument for surveys administered before and after the laboratory unit (mean ± standard error). Scores for the two major factors (preservation, utilization) and factor subscales are reported. Asterisks between bars indicate significant differences between surveys administered before and after the laboratory unit based on *t*‐tests

Students’ environmental perceptions based on their scores on both the PRE and UTL factors of the 2‐MEV scale were high on the preassessment, indicating that students had pro‐environmental perceptions before the start of the laboratory unit. Despite this, significant increases in pro‐environmental perceptions after the activity were observed for the PRE factor (*t*‐test *p* = .035). Average scores for PRE factor were 1.7 ± 0.5 on the survey administered before the lab unit, indicating a strong agreement with the importance of preserving nature (ecocentric viewpoint) and 1.5 ± 0.3 after the lab unit (Figure 3a), indicating a shift toward even more ecocentric views. This change was driven by a significant ecocentric shift in the “intent to support” subscale (Figure 3b; *t*‐test *p* = .032). While the “care with resources” (Figure 3c) and “enjoyment of nature” (Figure 3d) subscales of the PRE factor both shifted toward more ecocentric views, these differences were not significant. Average scores for the UTL factor on the survey administered before the lab unit were 3.9 ± 0.7, indicating a slight disagreement with using nature (anthropocentric viewpoint; Figure 3e) and 4.1 ± 0.6 after the lab unit, indicating a slight shift away from anthropocentric views (*t*‐test *p* = .061). There was a significant shift away from anthropocentric views in the “altering nature” subscale of the UTL factor (Figure 3f; *t*‐test *p *= .021). The “dominance” subscale of the UTL factor also exhibited a slight away from anthropocentric perspectives post‐laboratory unit (Figure 3g).

### 3.3 Frequency of study site visitation

**FIGURE 4:**
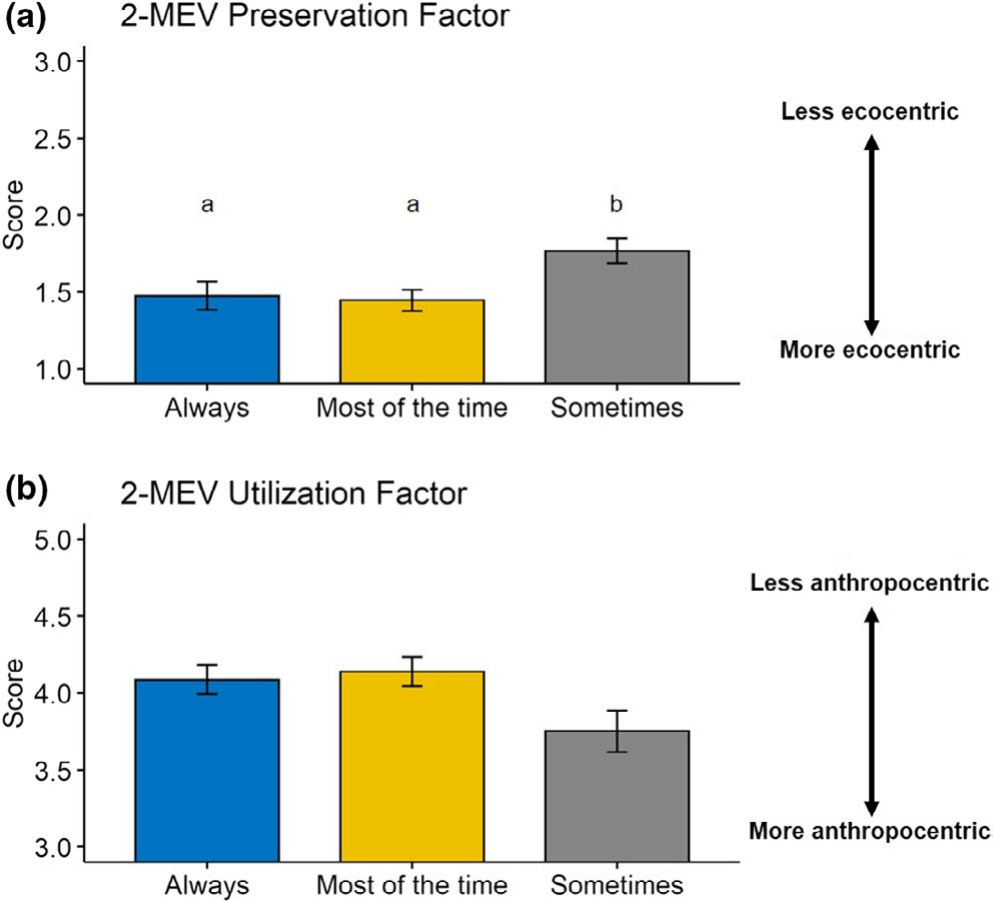
Scores on the 2‐MEV environmental perception survey for surveys administered after the laboratory unit (mean ± standard error). Student scores were separated based on self‐reporting of how often they visited their field site (always, most of the time, sometimes). Letters indicate significant differences between students who visited their field sites with different frequencies based on one‐way ANOVA

The amount of times students reported visiting their field sites over the course of the lab unit varied, with 33% reporting that they always visited their site, 46% reporting that they visited their site most of the time, 22% reporting they visited it sometimes, and 0% reporting that they never visited their field site. Field sites spanned the gradient from periurban to more rural (e.g., forest, shrubland, desert). For example, some students used their suburban yards as their field sites, while others visited local parks, and others ventured into nearby undeveloped areas. The number of times a student visited their field site had no effect on students’ understanding of the geographic region with highest biodiversity (ANOVA *F*_22,2_ = 0.99, *p* = .38; Figure 2a). However, the ability to explain the mechanisms responsible for the LDG after completion of the lab unit was higher in the groups of students who visited their field sites “always” and “most of the time”, although this difference was not statistically significant (ANOVA *F*_22,2_ = 1.13, *p* = .34; Figure 2b). The number of times a student visited their field site had an effect of environmental perceptions (Figure 4). For students who visited their field sites “always” and “most of the time”, the PRE factor scored significantly more ecocentric after the lab unit (PRE ANOVA *F*_22,2_ = 3.70, *p* = .03).

## 4 DISCUSSION

Knowledge of the geographic location of highest biodiversity can be viewed as lower order thinking, while the ability to explain the mechanisms of the LDG can be classified as higher order thinking (Bloom et al., 1956). Kern and Carpenter (1986) found that students participating in field studies exhibited increased levels of higher order thinking compared to those in the classroom. Many biology educators place high value on active learning and for ecology education, on outdoor ecological experiences (Barker et al., 2002; Jeronen et al., 2017; Randler, 2008) and PBE has been shown to increase ecological learning (Bögeholz, 2006; Brody, 2005). Fewer studies have focused on the benefits of field study to student learning at the undergraduate level (Boyle et al., 2007; Smith, 2004). For biodiversity education in particular, a reconnection with nature through education has been advocated (Lindemann‐Mathies & Bose, 2008; Scott et al., 2012). For example, undergraduate students across several majors (biology, ecology, coastal marine biology, environmental studies) conducting field‐based study learned aspects of biodiversity better than students studying biodiversity only within the indoor laboratory setting (Scott et al., 2012).

In our study, the student understanding of the lower order concept, where geographically biodiversity is the highest, was high prior to the start of the lab activity and while there was a small increase in the percentage of students answering this question correctly after the activity, this change was not significant. In contrast, higher order thinking, students’ ability to explain the mechanisms behind the LDG, significantly increased after the lab activity, with most students providing more well‐developed answers on the postlab survey. We posit several explanations for the high level of understanding prior to the lab activity. First, within the US currently, the link between biodiversity and human activity is part of the Next Generation Science Standards (NGSS Lead States, 2013) and biodiversity is one of the core ecological concepts in the Ecological Society of America's four dimensional ecology educational framework (Klemow et al., 2019). Thus, the vast majority of students in our class had prior experience with the LDG concept, even if they did not recognize the term at first pass. In addition, the population of students taking this lab course was primarily biology and environmental studies majors, students with a strong interest in science. Studies demonstrate that individuals with a background in biology have a greater understanding of the three elements of biodiversity (Lindemann‐Mathies & Bose, 2008). Therefore, their prior understanding of the LDG is not surprising.

Our prediction that pro‐environmental perceptions would increase after a NBE field study was supported. Despite high pro‐environmental perceptions prior to the lab unit, a significant shift toward pro‐environmental attitude was detected between pre‐ and post‐surveys. Increases in positive environmental attitudes in response to NBE has been reported in many studies across disciplines (Bunge, 2000; Karabinos et al., 1992; Kern & Carpenter, 1984; Sterling, 2009). Again, the population of students in this class may have influenced the high degree of pro‐environmental attitudes before the lab activity. Environmental literacy, pro‐environmental perceptions, and environmentally responsible behavior have been shown to correlate to college major (Ewert & Baker, 2001; Fusco et al., 2012; Isildar and Ylldrim, 2008). Hodgkinson and Innes (2001) found that although most university students exhibited pro‐environmental attitudes, attitudes among those majoring in biology and environmental fields were the most pro‐environment. Strong pro‐environmental perceptions before the lab activity may also have been due to the population of students in our study. The University of Portland is a regional university, with 75% of students coming from Oregon, Washington, and California. The majority of students in our study were from Oregon where a week‐long Outdoor School program is available to all students as part of the 5^th^ and 6^th^ grade curriculum. Thus, many of our students were exposed to NBE prior to the start of this course. Finally, the location of our University may have influenced pro‐environmental perceptions among students. The University of Portland is located in Portland, Oregon, a city considered to be a leader in promoting liveability, sustainability, and in preparing for climate change (Saha & Paterson, 2008; Slavin & Snyder, 2011) and thus students are continually exposed to pro‐environmental attitudes and behaviors on and off campus. In addition, the University of Portland itself promotes pro‐environmental attitudes via campaigns to curb food waste and a University Climate Commitment to be carbon neutral by 2040.

The increase in pro‐environment perception was stronger for the PRE factor of the 2‐MEV scale than it was for the UTL factor. All facets of the PRE factor showed higher ecocentric views after the lab activity, resulting in a significantly higher pro‐environmental score for this factor. In contrast, the UTL factor did not exhibit a significant shift in postlab activity, although the “altering nature” facet displayed a significant shift away from anthropocentric views. The 2‐MEV scale measures two distinct and not necessarily related aspects of pro‐environmental attitude: ecocentrism (PRE) and anthropocentrism (UTL) (Bogner & Wilhelm, 1996; Bogner & Wiseman, 1999). These two aspects of pro‐environmental attitude have been shown to be separate factors across ages, cultures, and geographic regions (Johnson & Manoli, 2011; Milfont & Duckitt, 2004). Further, studies have shown that shifts in scores on PRE and UTL factors after an intervention do not always happen in concert and can vary with gender, learning success, and learning achievement (Schumm & Bogner, 2016). Bogner et al. (2015) report that this may be due to the fact that someone might hold ecocentric views but still behave in an anthropocentric fashion.

We propose two possible explanations for the different behavior of the PRE and UTL factors related to our particular study: (1) the location of field sites and (2) the religious nature of our institution. For this lab activity, students were allowed to choose any local field site. Sites ranged from urban to undeveloped, with 83% classified as urban/periurban by participants. Human activities in urban ecosystems can have a positive impact on the environment and biodiversity, thereby complicating students’ perceptions of how human activities affect the environment (Tidball & Krasny, 2010). The only slight shift away from anthropocentric attitudes for the UTL factor postlab unit may relate to this phenomenon.

We did not specifically ask about religious affiliation in our survey and many students at the University of Portland do not align with the Catholic belief system. However, being at a Catholic institution where several courses on Christian traditions are part of the required curriculum may have influenced the trends in environmental perception. While there was a significant shift toward ecocentric on the PRE factor, the shift away from anthropocentric views on the UTL factor was much smaller. Traditionally, the Catholic worldview can be viewed as dualistic, with (hu)man(s) being separate and above nature and the care of nature being entrusted to (hu)man(s) (Binde, 2001). Consequently, the Catholic worldview has not been at the forefront of recognizing the human impact on nature. Although more recently, the *Laudato Si’* encyclical of Pope Francis (2015) recognizes the negative impacts that humans are having on the environment (Blay, 2019; Tan, 2019). Previous studies have demonstrated a negative correlation between religious beliefs and pro‐environmental attitudes and environmentally responsible behavior (Fusco et al., 2012; Greeley, 1993; Hand & Van Liere, 1984). For example, Fusco et al. (2012) found that students self‐identifying as Christian engaged in significantly lower environmentally responsible behavior. In a survey of 192 psychology majors at a university with similar characteristics as ours (private, Christian, liberal arts, in the US Northwest), all measures of environmental behavior were negatively related to Christian orthodoxy (Truelove and Joireman, 2009). As we did not specifically ask about religious affiliation in our survey, the different response in the 2‐MEV PRE and UTL factors warrants further examination.

Our prediction that improvements in ecological understanding and pro‐environmental perceptions would be highest for students visiting their field sites most often was supported. Student higher order thinking around the LDG concept and their pro‐environmental attitudes (PRE factor) were higher for students who visited their field sites “always” and “most of the time”. While many studies have demonstrated the positive effects of NBE on environmental literacy, few have focused on how the duration of the NBE experience affects learning outcomes. At the middle and high school level, studies have demonstrated that outdoor education and fieldwork improve ecological literacy across varying durations of the field experience, ranging from a single class period, to multiple consecutive days, to several sessions across multiple weeks (Bradley et al., 1999; Brody, 2005; Hiller and Kitsantas, 2014; Palmberg et al., 2015; Prokop et al., 2007). However, when comparing results of a specific program, duration may influence its success. For example, for primary and secondary students, a five‐day outdoor school experience was significantly more effective at promoting nature connectedness than a one‐day program (Braun & Dierkes, 2017). Less attention to the influence of duration of NBE on ecological literacy has been given at the undergraduate level. Undergraduate biology students attending a 10‐day residential field program demonstrated improved cognitive learning (Easton & Gilburn, 2012). In our study, there were no differences in student understanding of the lower‐order concept, where geographically biodiversity is the highest, but gains in student understanding were observed for the higher order question about mechanisms behind the LDG. This may be partially attributable to the reduction in cognitive load that comes from visiting the same location multiple times.

### 4.1 Study limitations

We found increased environmental literacy and shifts toward pro‐environmental perceptions after students completed a multiweek NBE study in an introductory biology lab course. However, we cannot be sure that the NBE experience itself was the cause or if these improvements resulted from the ecological content of the lab unit itself. Because environmental literacy was highest in students who visited their sites most often; we feel confident in attributing the increase between pre‐ and post‐activity surveys at least in part to the NBE. Another limitation of our study related to the voluntary participation in the field study portion of this lab unit. While students were strongly encouraged to visit their field sites multiple times, they had the option to use data available online when they were unable or unwilling to visit their field sites. While most students visited their field sites at least some of the time (0% of students self‐reported never visiting their field site), we would expect that the students who opted to visit their field sites more often may have been the students most interested in ecology and the environment a priori. Thus, it is possible that the more pro‐environmental attitude in this group post‐activity may be partially attributable to the population of students most likely to visit their field sites. Finally, we would like to acknowledge that the field study approach used in this lab activity has the potential to result in a noninclusive learning experience for certain students. Not all students were able to visit field sites, due reasons such as Covid‐19 restrictions, safety, lack of transportation, etc. In particular, students residing in Hawaii were most impacted by Covid‐19 limitations as the state entered a second stay at home order for more than one month during the lab activity, limiting the participation of these students to their home yards as the field site. While we strongly encouraged all students to go outside and collect data, even if it was from a location as urban as a parking strip, a few students opted to use completely online resources to complete parts of this unit. As student learning was highest for students willing or able to visit their field sites most often, this activity may have created an inequitable learning environment. When utilizing this approach to ecological field study in the future, every effort should be made to enable every student to participate in the authentic field experience.

## 5 CONCLUSION

Our results provide further evidence as to the value of NBE for the introductory biology laboratory, even in settings where students are conducting their field activities without direct supervision. This lab activity was formulated as a consequence of the shift to online education in response to the Covid‐19 global pandemic. Since the design of this course, several papers have been published tackling the challenges of conducting ecological labs online and outlining ways to leverage these challenges to improve pedagogy, student learning, and student retention in ecology and environmental fields through strategic course redesign (e.g., Bacon & Peacock, 2020; Cooke et al., 2021; Harris et al., 2020; Paudel, 2021). In our study, a half‐semester long NBE lab unit where students utilized local field sites improved both ecological literacy and pro‐environmental perceptions, particularly in students visiting their field sites on multiple occasions. Our findings suggest that high‐impact field studies are possible even in an online learning format.

The lab unit described in this study provides a potential approach to teaching ecology in an online setting that can easily be adapted to fit the needs of a particular curriculum. For example, we modified this activity for our return to in‐person instruction in spring 2022. The class will visit two different types of field sites (urban forest, wetland) as a group. Soil collection and analysis, vegetation surveys, and bird observations will be conducted at each site over a four‐week period. For the final project, students will work in small groups to compare their data with data generated from other geographic areas in the previous year when students completed the lab activity remotely. As it is likely that no one in the group will have firsthand knowledge of the ecology of these other areas, students will also spend time researching the natural history and ecology of these areas prior to completing the final LDG synthesis activity. As an alternative or in addition to using data generated in previous years, students could use online resources, such as, iDigBio (https://www.idigbio.org/), iNaturalist, or EARTHDATA (https://earthdata.nasa.gov/), to generate diversity data from other locations for comparison. As access to digital species occurrence data increases, there is ample opportunity to adapt this lab to fit the needs of online, in‐person, or hybrid education.

## CONFLICT OF INTEREST

The authors have no financial or nonfinancial interests that are directly or indirectly related to the work submitted for publication.

## AUTHOR CONTRIBUTIONS

**Christine L. Weilhoefer:** Conceptualization (lead); Data curation (equal); Formal analysis (lead); Methodology (equal); Project administration (lead); Writing – original draft (lead); Writing – review & editing (equal). **Sarah Schmits:** Conceptualization (supporting); Data curation (equal); Methodology (equal); Project administration (supporting); Writing – review & editing (equal).

## Data Availability

The dataset generated during and analyzed during the current study can be found at: Weilhoefer, Christine (2022), Weilhoefer_Schmits_2022, Dryad, Dataset, https://doi.org/10.5061/dryad.4b8gthtf7.

## APPENDIX 1

Schedule of weekly lab activities for the 8‐week lab unit

**Table:**

Week	Topic	Location
1	Field site selection and transect establishment	Field site
2	Soil core collection	Field site
3	Soil analysis for pH, nitrogen, phosphorus, potassium	Home lab
4	Vegetation survey	Field site
5	Examining the relationship between vegetation diversity metrics (Shannon diversity, species richness) and soil properties: bar charts, boxplots, t‐tests	Home lab
6	Bird observations and calculation of bird diversity metrics	Field site
7	Site history research	Home lab
8	Latitudinal diversity gradient synthesis activity	Small groups

## APPENDIX 2

Flow of a typical weekly lab activity indicating activity type, type of instruction (synchronous or asynchronous), and estimated time for completion

**Table:**

Step	Activity	Time
1	Pre‐lab introduction and reading ‐ asynchronous	0.5–1 h
2	Lab methods demonstration ‐ synchronous with instructor	0.5–1 h
3	Field site visit (weeks 1, 2, 4, 6) ‐ asynchronous	1–2 h
	Lab work (weeks 3, 5, 7) ‐ asynchronous	
4	Observation synthesis and reflection ‐ asynchronous	0.5–1 h

## APPENDIX 3

Questions from the ecological concept survey administered before and after the laboratory unit and ancillary information collected at the end of the laboratory unit

**Table:**

Ecological concept survey	1. In which geographic area is biodiversity the highest? (choices: tropics, mid‐latitudes, polar regions)
	2. Provide 3 factors that can increase the biodiversity of an area. (free response)
Ancillary information	1. What is your lab section? (choices: A, B, C, D)
	2. In what state are you located? (free response)
	3. How often did you visit your field site (choices: always, most of the time, sometimes, never)
	4. How would you classify your field site along an urban gradient? (choices: urban, periurban, rural)
	5. Which habitat type best describes your field site? (choices: forest, grassland, desert, wetland, urban park, rural park, other)
